# Radiologic Evaluation of Small Renal Masses (I): Pretreatment Management

**DOI:** 10.1155/2008/415848

**Published:** 2009-03-29

**Authors:** A. Marhuenda, M. I. Martín, C. Deltoro, J. Santos, Jose Rubio Briones

**Affiliations:** ^1^Departamento de Radiología, Instituto Valenciano de Oncología, C/Profesor Beltrán Báguena 8, 46009 Valencia, Spain; ^2^Departamento de Urología, Instituto Valenciano de Oncología, C/Profesor Beltrán Báguena 8, 46009 Valencia, Spain

## Abstract

When characterizing a small renal mass (SRM), the main question to be answered is whether the mass represents a surgical or nonsurgical lesion or, in some cases, if followup studies are a reasonable option. Is this a task for a urologist or a radiologist? It is obvious that in the increasing clinical scenario where this decision has to be made, both specialists ought to work together. This paper will focus on the principles, indications, and limitations of ultrasound, CT, and MRI to characterize an SRM in 2008 with a detailed review of relevant literature. Special emphasis has been placed on aspects regarding the bidirectional information between radiologists and urologists needed to achieve the best radiological approach to an SRM.

## 1. INTRODUCTION

Over the last 3 decades, there has been a rising trend to define small renal masses (SRMs) as masses below 4 cm in diameter [1], making it the major reason for the 126% increase in incidence of renal cell carcinoma (RCC) in the United States. The reason for that is well known; the increasing number of imaging examinations performed for unrelated indications with many renal neoplasms of small size and early stage incidentally detected. Faced with this situation, urologists do not only suggest surgery as 30 years ago, but also offer different options to deal with the problem. Most of their decisions are based on radiological characterization of the SRM, as biopsies of these masses have not been completely accepted by the international urological community.

So, evidently urologists have to ask their colleagues in the Radiology Department to improve their explorations expecting more and more extensive radiological reports analyzing not just the presence of the mass. The SMR analysis must be carried out by both a radiologist and an urologist, as bidirectional information is extremely important to define the most probable nature of the mass.

The accurate diagnosis of a renal mass depends on many factors, including the clinical history; so there is some clinical information that urologists have to report to their radiologists:

- presence of a familial syndrome,
- presence of a urological tract infectious disease previous or concomitant to the diagnoses of the SRM,
- presence of previous stone disease and related treatments,
- presence of previous renal trauma,
- presence of kidney disease and renal insufficiency.

A high-quality imaging examination, under the control of a radiologist, is essential. The most accurate diagnosis of a renal mass is then made according to the nature of the imaging findings, the experience of the radiologist, and the quality of the examination, as well as the exclusion of conditions that can mimic a renal neoplasm. There are some key points that, due to their therapeutic decision-making importance, radiologists need to provide in their reports:

- signs suspecting fat involvement in an SRM,
- metabolic behavior during the different phases of CT and MRI after contrast administration, allowing to characterize benign SRM,
- the need (or not) to complete studies with different techniques,
- accurate and standard (for followup in case of watchful waiting policy) measurement of 3 diameters of the SRM,
- signs of active tumoral tissue after conservative treatments which do not remove the SRM,
- differential diagnosis of residual tumour with complications after partial nephrectomies and foreign bodies used to achieve haemostasis.

Having established the collaboration between urologist and radiologist for this review paper, the aim of the two complementary chapters submitted for the SRM diagnoses and characterization is to give some light on the new challenges which face radiologists nowadays, extremely important for the SRM management. 

## 2. OBJECTIVES

Renal cell carcinoma and oncocytoma are indistinguishable from each other at imaging. Many other renal lesions must be considered, such as angiomyolipoma (AML), lymphoma, metastatic disease, renal anomalies, and other pseudotumors that can mimic renal cell carcinoma. Although it is possible to make this differentiation by using the imaging findings alone, the clinical history can often be very important in making the correct diagnosis. In fact, before making a diagnosis of renal cell carcinoma, one should be certain that none of these possible mimickers of renal cell carcinoma are potentially present.

Staging by TNM system can be considered a prognostic classification, and there is evidence that the smaller the size, the better the prognosis [2, 3] . The increasing incidence of renal mass manifestations of tumours that are confined to the renal capsule and relatively small in size has stimulated a growing trend toward nephron-sparing surgical techniques, as current data show survival rates comparable to those associated with radical nephrectomy.

Imaging findings that can affect the decision to perform partial nephrectomy included tumor size in three planes: tumor location within the kidney; presence of a pseudocapsule (a thin band of fibrous tissue and compressed renal parenchyma surrounding the lesion); tumor invasion of the renal sinus fat, collecting system, renal vein, or perinephric fat; presence of lymphadenopathy; morphologic and physiologic status of the contralateral kidney. All these aspects are evaluated by means of different imaging techniques.

The increased implementation of kidney-sparing surgery for renal cell carcinoma may create an important role for diagnostic imaging in the discovery of small synchronous carcinomas. Radiologist should be aware of the possibility of tumor multifocality or of adrenal metastases from a high-grade small renal tumor as well as of the association of RCC with lymphoma [4].

The challenge is to detect and delineate all lesions to ensure complete surgical excision while preserving the maximal amount of functioning parenchyma. For patients who are not surgical candidates, imaging staging, along with the other factors, can provide prognostic information.

## 3. ULTRASOUND

The fact that renal neoplasms have been detected earlier and with increased frequency is well documented in the literature [5, 6]. This is probably due to two major factors. One factor is that there has been a considerable increase in the number of people who undergo kidney imaging in the general population because of the widespread use of ultrasound (US). The other reason is that this imaging technique is able to depict lesions of the kidneys that could be missed with urography [7]. This increased detection of renal neoplasms also results in the increased detection of benign lesions and nonneoplastic masses, particularly renal cysts. Therefore, the differentiation between a neoplastic and a nonneoplastic lesion is a common dilemma.

To differentiate benign from malignant SRM can prove even more problematic because the findings can also become smaller, hence requiring more detailed and more sensitive imaging studies.

**Figure 1:**
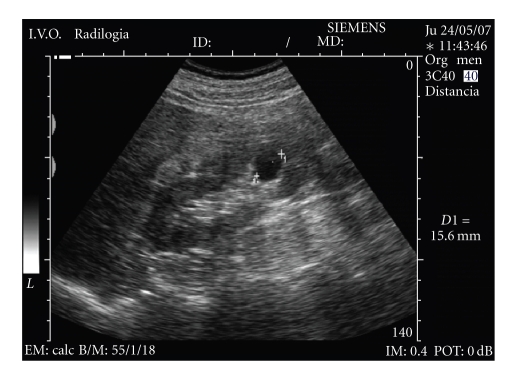
Simple cyst as anechoic lesion with a sharply defined back wall and enhancement of through sound transmission.

Ultrasound plays an important role in the detection and evaluation of these SRMs. While this technique may not be as sensitive as contrast-enhanced CT or MR for revealing SRM, US has been the initial technique in the discovery of a large number of these incidentally discovered tumors when the kidney is studied in the course of abdominal imaging. Sonography is very accurate in distinguishing liquid from solid tissue. Therefore, its major use in these small lesions is to help differentiate small cysts (see Figure 1) from small solid tumors [8]. Maintaining rigid criteria is necessary to maintain the high accuracy possible with this technique.

**Figure 2:**
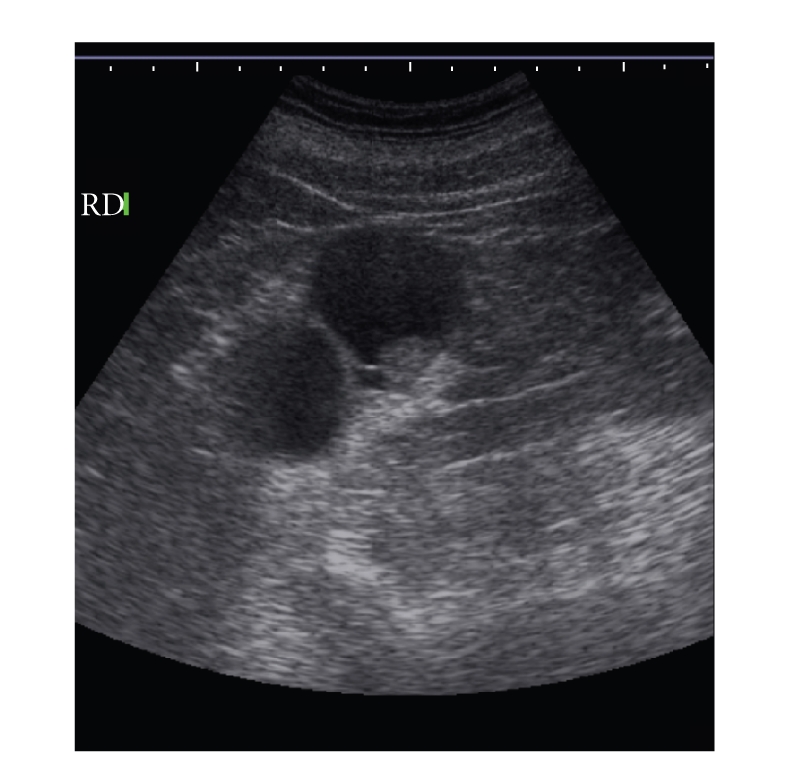
A cyst with nodular thickening of the wall and internal septa.

In the general population, renal cysts are the most common space-occupying lesions in the kidney. With this technique, 80% of detected renal masses are characterized as simple cysts [9] thus ending their diagnostic evaluation. The remaining 20% of renal masses require further study with CT or MR imaging [10]. Any mass detected that does not meet the strict sonographic criteria for a simple cyst should be further evaluated with CT or MR imaging of the kidneys. However, one or two thin septations may also be visible sonographically in simple renal cysts [11]. Because these findings are diagnostic, no further imaging or followup is needed in the evaluation of these lesions. However, other atypical features sonographically detected calcifications; more than two septations, septal thickening or nodularity, and the presence of solid components indicate that sonography alone will not be adequate for complete evaluation of these renal masses (see Figure 2). The addition of Doppler sonography, color Doppler sonography, power Doppler sonography [12, 13], and sonographic contrast agents may further improve the detection and characterization of renal masses. However, none of these techniques preclude the need for CT or MR imaging of renal masses that do not meet the sonographic criteria for diagnosis of a simple cyst.

**Figure 3:**
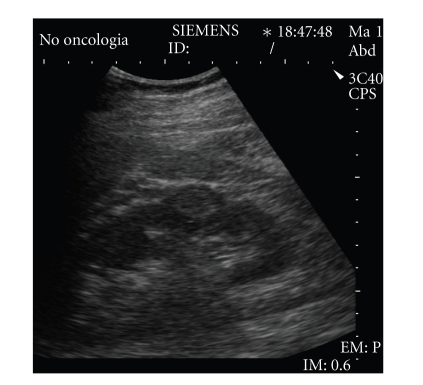
Well-defined hyperechoic small renal mass. Pathologic analysis of the surgical specimen revealed a renal cell carcinoma.

In the study of solid renal masses, the role for US has been mainly centred on the differentiation of RCC and AML, which are the most common malignant and benign solid renal tumors, respectively [14–17]. When a solid mass is diagnosed, RCC or AML should be initially considered because of the high frequency of their occurrence. At US, most AML lesions are markedly hyperechoic relative to renal parenchyma. They may appear less echogenic depending on the relative proportion of fat, smooth muscle, vascular components, and haemorrhage in the lesion [18, 19]. RCC displays a broad range of echogenicities. Although often thought of as hypoechoic or isoechoic, recent studies have shown that most RCC are hyperechoic relative to renal parenchyma and that up to 12% simulate AML [14–17]. Forman et al. [14] have shown that one third of small RCC are as echogenic as a “classical” AML. An echotexture equal to that of renal sinus fat seen in a small renal mass is, therefore, no longer considered adequate to exclude the diagnosis of malignancy (see Figure 3).

**Figure 4:**
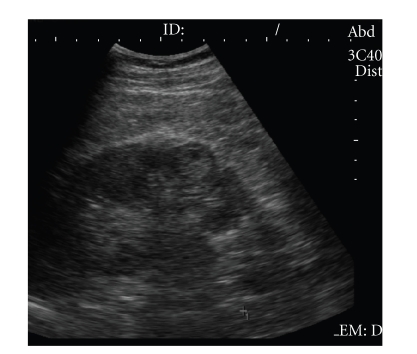
Well-defined hyperechoic small renal mass with hypoechoic rim and intratumoral cystic area, confirmed with pathologic analysis as renal cell carcinoma.

Other ultrasound signs have been used to differentiate between hyperechoic RCC and AMLs. The presence of an anechoic rim and/or an intratumoral cyst is only seen in RCC (see Figure 4). The presence of acoustic shadow is specific of AML. However, the detectability of these findings varies [15, 16], their diagnostic value has not been established, and the presence of these features is not sufficient to differentiate RCC from the other solid renal masses that are incidentally detected on gray-scale US. On the power Doppler US, the analysis of the vascular distribution has not increased the diagnostic accuracy for small renal tumors [12]. Contrast-enhanced Doppler US can increase the detection of intratumoral vascularity compared to color/power Doppler US [20]. However, their signal intensity has not been found to be sufficiently intense for tumor characterization. Recently, the development of contrast-enhanced harmonic US imaging has provided a better assessment of the diagnostic accuracy of RCC as compared with gray-scale US by allowing better visualization of the intratumoral anechoic areas and the pseudocapsule than can the gray-scale US [21], but there still exists an overlapping of signs of RCC and the other solid renal masses, making it necessary to use CT or MR imaging in the study of small renal masses.

## 4. CT

Helical CT is generally accepted as the critical imaging test for the classification of renal masses. Radiation exposure is the greatest disadvantage of this technique. MRI is comparable to helical CT for detection, diagnosis, and staging of renal masses. However, CT has the advantages of widespread availability, shorter examination time, and lower cost in comparison with MRI.

A detailed analysis of a variety of CT features is required, including the size, location, appearance on unenhanced scan, the presence and location of calcifications, the presence and size of a cyst wall or septations, and the amount and pattern of contrast enhancement [7, 22, 23].

### 4.1. CT technique

Single detector and especially multidetector spiral (MDCT) have refined the diagnostic evaluation of renal pathologic conditions. Compared with single-detector helical CT, MDCT allows the kidneys to be scanned with a collimation of less than 5 mm during a single breath hold [24]. From a single data set obtained with thin collimation, both thin and thick sections can be reconstructed and no additional radiation exposure is required to obtain the thin sections. This dataset is manipulated by using a workstation to produce volume-rendered and three-dimensional (3D) images when necessary. The 3DCT images can be viewed in multiple planes and orientations to define the lesion.

A triphasic imaging protocol consists of an unenhanced phase through the kidneys, an arterial or corticomedullary phase through the liver and kidneys (between 25 and 70 seconds after the start of injection of contrast), and a portal venous or nephrographic phase of the entire abdomen (between 80 and 180 seconds). Excretory phase (>180 seconds) is occasionally helpful.

An initial series of unenhanced scans provides a baseline from which to measure the enhancement within the lesion.

The corticomedullary phase is useful to perform 3D reconstructions and to depict the renal vasculature. Furthermore, this phase is considered essential for staging.

The nephrographic phase provides greater lesion detection and improved lesion characterization of renal masses than corticomedullary phase [25, 26]. However, a case of renal cell carcinoma visible only during the corticomedullary phase has been shown in the literature [27]. The excretory phase is occasionally helpful to better delineate the relationship of a centrally located mass within the collecting system. Delayed scanning (15 minutes) can also be used in lieu of unenhanced scanning to characterize an incidental renal lesion detected on a routine contrast-enhanced CT scan [28].

**Figure 5:**
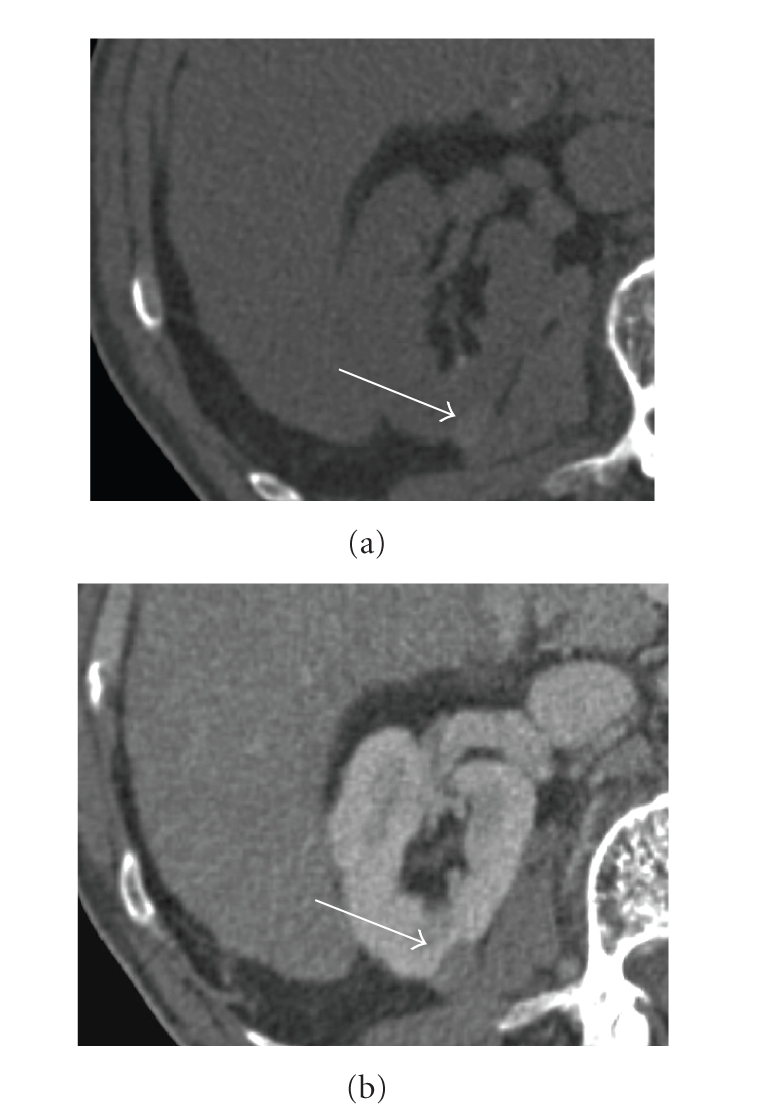
Hemorrhagic cyst. (a) Unenhanced CT scan shows a hyperattenuating small renal mass (62 H) (arrow). (b) Contrast-enhanced CT scan during the nephrographic phase reveals light enhancing (attenuation value increased 9 H:71 H) (arrow).

At present, there is no worldwide agreement upon the specific number that can be used as definitive and unequivocal evidence of enhancement within a renal mass, and it has been proposed by many authors that the previously used threshold of 10 HU should be increased to 20 HU (a currently accepted criterion) (see Figure 5). Some authors think that a renal mass that enhances 10–20 HU is indeterminate and needs further evaluation [29].

### 4.2. Imaging of specific small renal masses

#### 4.2.1. Cysts

Most small renal masses incidentally discovered on CT are simple cortical cysts that need no further evaluation. The Bosniak classification system is used to asses the likelihood of malignancy in cystic renal masses on the basis of lesion complexity in CT imaging [30, 31]. Application of the Bosniak classification can be difficult in the case of an indeterminate renal lesion, especially if it is small. Bosniak admits that distinction between category II (not requiring surgery) and category III (requiring surgery) can be very difficult [32].

#### 4.2.2. Angiomyolipoma

**Figure 6:**
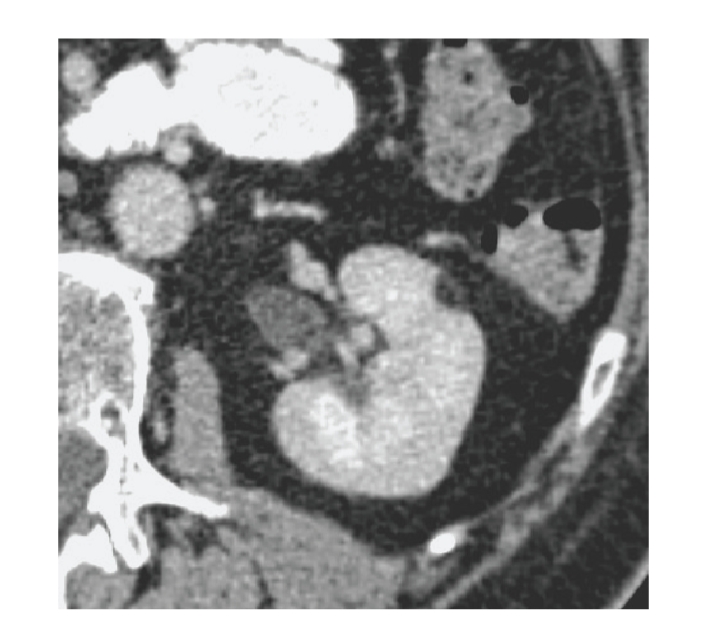
Angiomyolipoma. Contrast-enhanced CT shows a small homogeneous fat-containing mass.

It is typically a solid lesion that exhibits fat density on CT scans (−10 to –100 HU) (see Figure 6). However, in some cases, it may contain very small quantities of fat that can be overlooked. Angiomyolipomas rarely contain calcification, and, therefore, a diagnosis of angiomyolipoma should not be made if a lesion contains fat and calcium [33, 34]. There have been few case reports of fat from RCC that also contain calcification. In such cases, a renal cell carcinoma must be considered the most likely diagnosis.

#### 4.2.3. Oncocytoma

**Figure 7:**
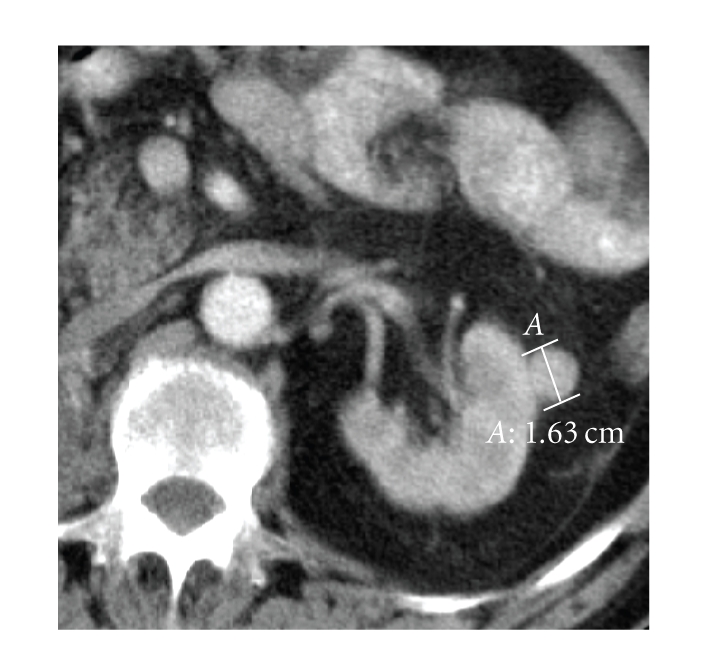
Oncocytoma. Small mass isoattenuated to renal parenchyma after contrast.

It is usually a hypodense mass, homogeneous, with smooth contours and a tendency to enhance avidly (see Figure 7). Until now, an oncocytoma was suggested on postcontrast CT by the presence of a central hypoenhancing scar. Because of its lack of specificity, patient management has been unaffected by the presence of this finding. Renal cell carcinoma and oncocytoma are indistinguishable from each other at imaging.

#### 4.2.4. Renal cell carcinoma

**Figure 8:**
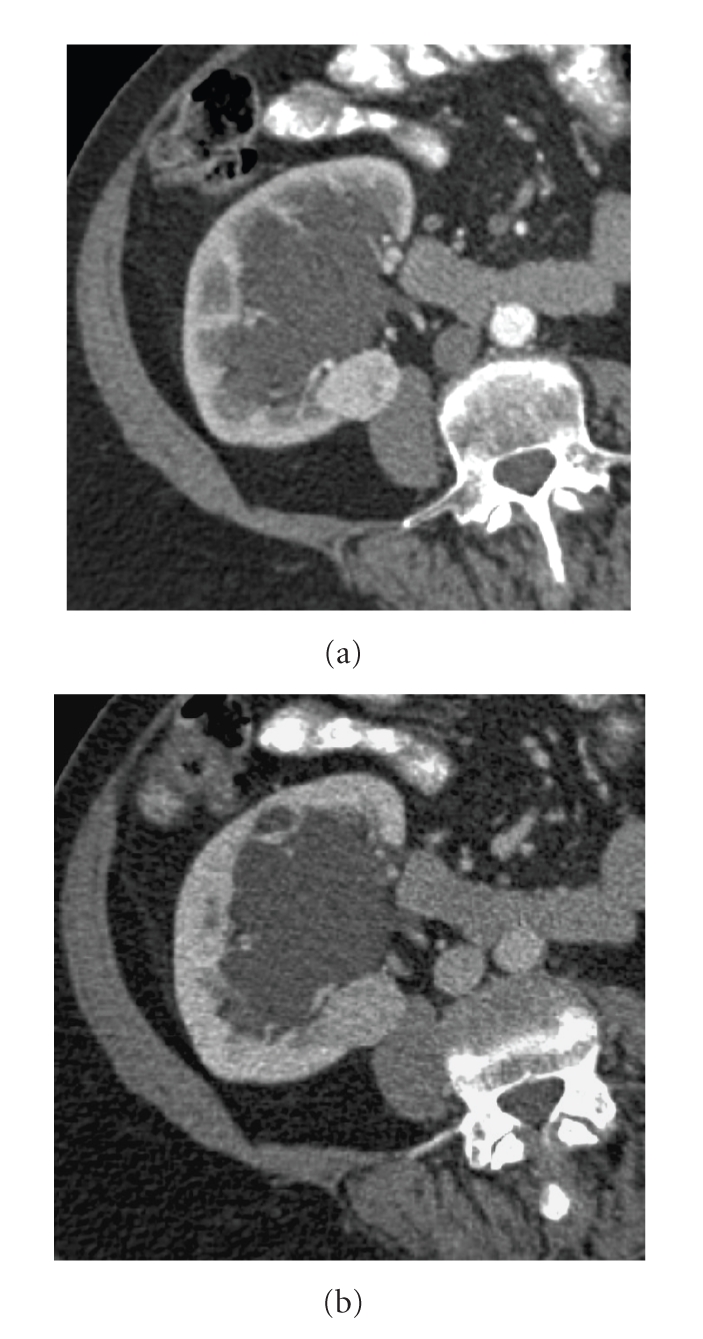
Small hyper vascular renal cell carcinoma. (a) Contrast-enhanced CT shows small renal mass that enhances early in corticomedullary phase. (b) Rapid washout in nephrographic phase.

The imaging characteristics of RCC are extremely varied, with masses ranging from cystic to solid, from homogeneous to heterogeneous and necrotic, from small to large, and from localized to extensive. The typical CT appearance of small RCC is a homogeneously isodense/hypodense mass, noncalcified, with an attenuation value of 20 HU or more, that enhance avidly and early with contrast medium (see Figure 8) [23]. The early-stage contrast enhancement is believed to be caused by tumor angiogenesis. However, a small proportion of RCC are hypovascular, and the amount of enhancement may be minimal. Small RCC with a predominantly cystic growth pattern, necrosis, or calcifications (peripheral curvilinear or punctate central) are uncommon. Areas of fat attenuation can be present within renal cell carcinomas, but are uncommon in small tumors.

Furthermore, a small RCC can be hyperattenuating. If the lesion is depicted only on enhanced CT, delayed scanning can also be used. Macari and Bosniak [28] have suggested that measurement of the washout of contrast material from at least 15 minutes allows differentiation between hyperdense cyst and renal neoplasms. The washout of 15 HU or more indicates that, excluding vascular abnormality, the mass is solid. A lack of washout indicates that the mass is probably a hyperattenuating cyst.

**Figure 9:**
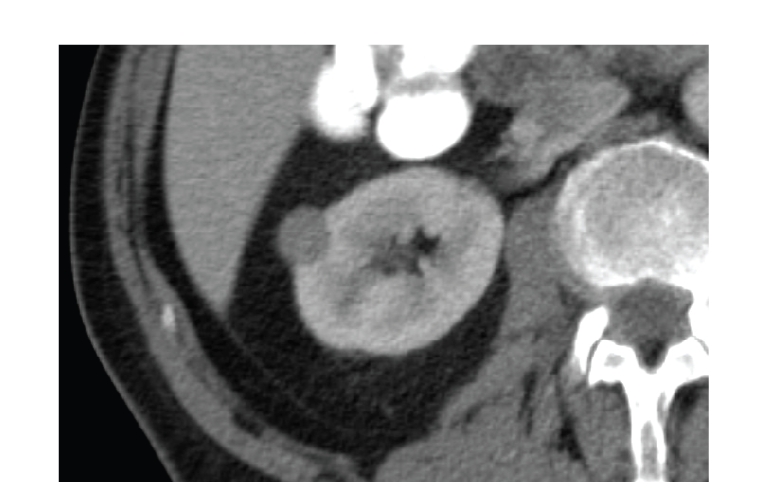
Papillary renal cell carcinoma. Contrast-enhanced CT shows small homogeneous mass that is mild and less enhanced than renal parenchyma does.

On the other hand, renal cortical tumors are family neoplasms with distinct cytogenetic and molecular characteristics and varying malignant potential. In the 1997 Heidelberg classification, renal cell carcinoma was subdivided in subtypes [35]. It has been suggested that certain imaging features may be associated with different subtypes of solid renal cortical tumors [36, 37]. The most consistent finding in these studies was that the degree of enhancement was the most valuable parameter for differentiation of RCC subtypes, as clear cell RCC enhance to a greater degree than other subtypes, especially papillary RCC. Clear cell RCC is also strongly associated with a mixed enhancement pattern of both enhancing soft-tissue components and low-attenuation areas (necrotic or cystic changes). When homogeneous or peripheral enhancement is present, clear cell RCC is a less likely diagnosis, and other cell types should be considered. Notably, a majority of papillary tumours were either homogeneous or peripheral enhancement (see Figure 9). The presence to neovascularity was mildly associated with more aggressive tumor. The clear cell RCC (and oncocytomas) enhanced avidly during the parenchymal phase; the chromophobe RCC (and lipid poor angiomyolipoma) enhanced moderately and papillary RCC enhance mildly.

#### 4.2.5. Non-Hodgkin's lymphoma

Lymphoma can have a variable appearance and may on occasion resemble renal cell carcinoma. Most frequently, it manifests as bilateral solid renal masses, and in a patient with systemic lymphoma, the proper diagnosis is not difficult. Characteristically, lymphoma often infiltrates into the kidney via the renal sinus or surrounds the kidney. In a patient with known systemic lymphoma to whom a renal mass with imaging is detected, systemic treatment for lymphoma should be instituted. If the patient's systemic disease responds, and the renal mass does not respond, biopsy of the mass is indicated. However, lymphoma may rarely manifest as a solitary renal mass or a homogeneous infiltrating renal mass. In this case, biopsy of the mass is indicated previous to systemic therapy.

#### 4.2.6. Metastases

Metastatic disease to the kidney typically manifests as multiple bilateral renal masses, often associated with metastatic disease to other organs. They are often poorly defined and infiltrate the renal parenchyma. With the appropriate clinical history, the diagnosis is straightforward. However, in a patient with a solitary renal mass (especially an infiltrating mass) and history of previous malignancy, percutaneous renal biopsy is indicated for a definitive diagnosis.

#### 4.2.7. Benign mesenchymal tumors

Included leiomyomas, lipomas, fibromas, and mixed mesenchymal nodules. They are usually small (<1 cm) lesions, found in autopsies.

#### 4.2.8. Pseudotumors

They include congenital anomalies (prominent renal columns of Bertin, renal dimorphisms, and dromedary humps) and acquired pseudotumors (hypertrophied normal renal parenchyma). This condition enhances identically to the normal renal parenchyma. In these situations it proves appropriate to scan during the corticomedullary and nephrographic phase.

#### 4.2.9. Renal mass mimickers

These include inflammatory masses (including focal pyelonephritis, renal abscess) and hematoma. A careful evaluation with high-quality CT or MR examination combined with the clinical context of the case and a familiarity with this group of “lesions” should reveal its true nature. Most hematomas are perinephric and are surrounded by fat stranding. They may occasionally have a masslike appearance, and some may not be discovered until long time after the traumatism. Chronic hematomas can have calcifications and do not enhance.

### 4.3. Diagnosis and management of renal call carcinoma with MD-CT

CT remains the most widely available and single most effective modality for staging renal call carcinoma [38, 39]. 3DCT combined with CT angiography has the potential to provide all the critical information needed to plan the surgical procedure. 3DCT images can be viewed in multiples planes and orientations to define the tumor and its relationship to the renal surface, the collecting system, and adjacent organs. A 3DCT angiogram can be created to delineate the renal arterial and venous anatomy.

The anatomic extent of the tumor at the time of diagnosis is the single most important factor in determining prognosis (see Table 1) [38].

Most urological surgeons continue to refer to Robson's classification, which is essentially a surgical staging approach. This system includes the important staging variables that have survived scrutiny over the years. Confinement within the renal capsule, penetration into the perirenal fat, invasion into the renal vein, and lymph node metastases are all important in determining the prognosis.

Under and overstating of perinephric invasion are the most common staging errors at CT [40]. The most specific finding of stage T3a, the presence of an enhanced nodule in the perinephric space, is highly specific but also low sensitive. The differentiation between stage T2 and T3a tumors is very problematic.

**Figure 10:**
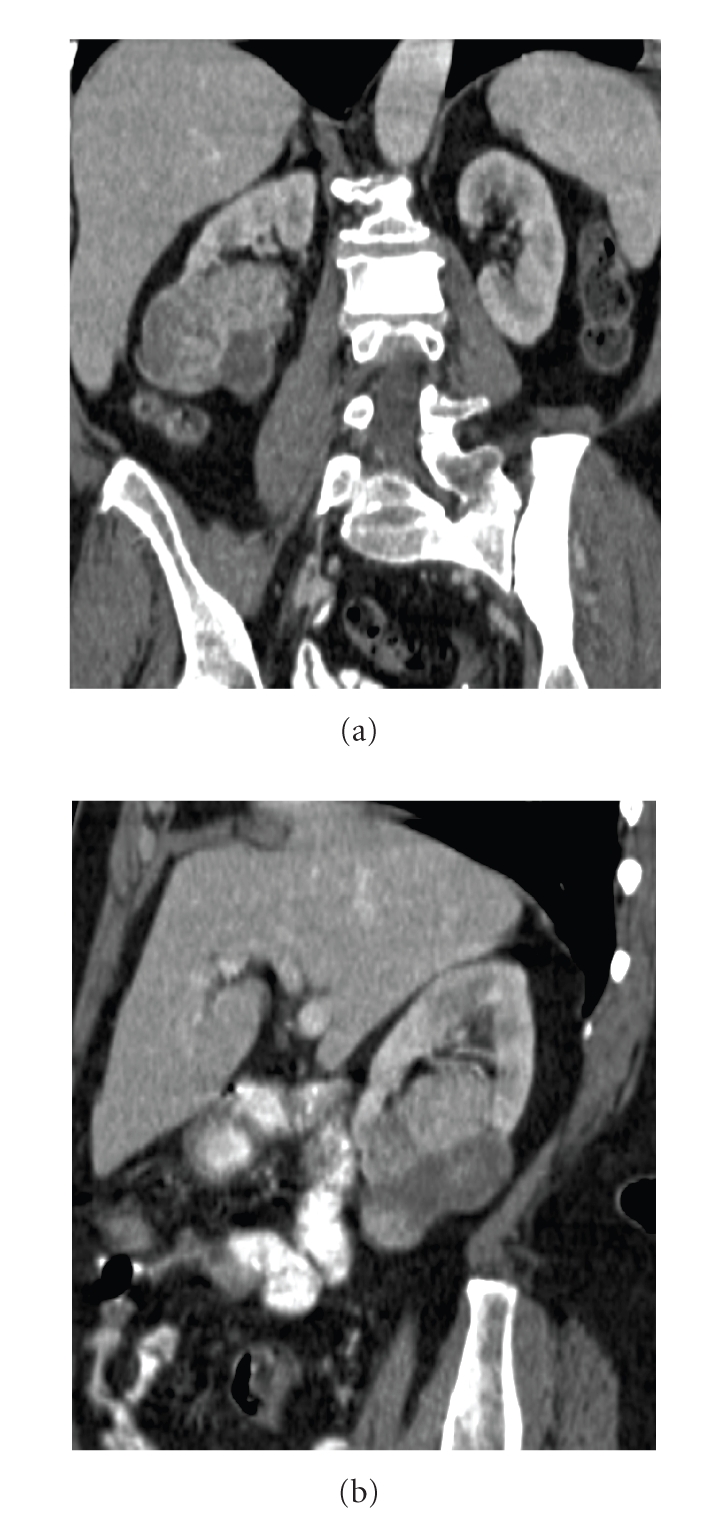
Renal cell carcinoma. (a) Three-dimensional CT with coronal reconstruction, (b) sagittal oblique reconstruction.

If tumoral spread within the IVC is identified, precise delineation of the superior extent of the thrombus is essential for the surgeon to plan the optimal surgical strategy for thrombectomy and minimize the risk of embolism. The level of involvement of the IVC dictates the surgical approach. Involvement of the IVC is best shown during corticomedullary phase. Because of its multiplanar capability, magnetic resonance imaging is the preferred modality to image. However, the three-dimensional CT with sagittal and coronal reconstructions is also effective in depicting the superior extent of inferior vena cava thrombus (see Figure 10), with the advantages of widespread availability, shorter examination time, and lower cost in comparison with MR [40].

## 5. MR

### 5.1. Indications

Although ultrasound and CT are often combined to reveal and characterize most renal lesions, MR is sometimes required when indeterminate lesions are found [41], or when a hyperattenuating renal mass is observed on CT [42]. Furthermore, some renal masses are incidentally discovered when MR imaging is performed to answer questions other than urology ones.

On the other hand, MR imaging has been considered the most accurate method for those patients with contraindications to iodinated contrast administration such as in patients with renal failure and those with an allergy to iodinated contrast material [43].

Recently, nephrogenic systemic fibrosis was linked to gadolinium contrast agent exposure in patients with renal failure; therefore its use should be reserved for neurological and vascular cases where the quality of information gained was sufficient to justify the risk of potential devastating adverse effects [44]. Nevertheless, the better contrast resolution of MR imaging, as opposed to CT, is an undoubtful advantage in those patients for performing an MR exploration, even though no contrast is provided.

MR imaging has been considered useful not only in characterization but also in evaluation of most cystic renal masses with the Bosniak classification system [45]. In addition, MR imaging can potentially improve staging and preoperative studying of a renal mass [41, 46–51] as well as tumor multifocality, collecting system invasion, and venous invasion [52]. Moreover, MRI is capable of showing the patency of a blood vessel without the use of intravenous contrast medium.

The accuracy in detection and characterization of SRM carries great significance when nephron sparing surgery is being considered for a renal cell carcinoma because synchronous lesions measuring 1–15 mm have been reported to a frequency as high as 19.7% [53].

### 5.2. Basic technical examination

A proper technical examination is needed for studying a renal mass which is indeterminate after US and CT techniques. Owing to intrinsic proprieties of magnetic resonance examination, high-quality renal MR imaging is dependent on multiple factors, includingpatient cooperation in holding breath. In patients referred for evaluation of a renal mass, examinations are currently performed with a torso phased-array coil, preferably during a more reproducibility end-expiratory breath hold [29, 54].

Different protocols can be used by different institutions depending on their technical requirements. Essentially, the common aspects are shown as follows. Prior to contrast administration, sequences are used to obtain images weighted on T1 and T2, in and out of phase T1, and a precontrast 3D weighted T1 with fat saturation can be acquired in different planes, basically on axial plane but also coronal or sagittal can be employed to best depict the mass, especially for such lesions located in renal poles. This approach is most important when evaluating a patient with a solitary kidney that contains a renal neoplasm that is amenable to partial nephrectomy.

These sequences provide us with morphologic information about the renal parenchyma and a renal mass (location, size, and signal intensity), parenchymal structures and adjacent organs, vascular structures and lymphadenopathies, and are designed to improve visualization of tisular intrinsic characteristics (fluid, haemorrhage, fat, fibrosis), except for detecting calcification.

Use of gadolinium as a contrast agent has been described as a higher detection and characterization of SRM with MR imaging to a higher level than does contrast CT [55, 56]. After contrast administration, MR angiography, MR venography, and MR urography are performed by using an oblique coronal breath-hold 3D fat-suppressed dynamically acquired T1-weighted spoiled sequence. The imaging delay for MR angiography is based ona bolus test with a power injector. MR venography and MR urography are performed at approximately 30 seconds and 5 minutes after MR angiography, respectively. The postcontrast acquisition is performed between MR venography and MR urography. For the characterization of renal masses and to determine the presenceor absence of enhancement, most authors recommend an imaging delay of 3–5 minutes [29].

These sequences are used to increase conspicuity of a renal mass, and to depict the renal arteries and veins, for the evaluation of the extent of the tumor in the perinephric fat and the relationship of a renal tumor to the hilar vessels and collecting system, which is helpful to the urologist in surgical planning [49].

### 5.3. Important features to recognize on MR imaging

#### 5.3.1. Signal intensity

Most renal masses are hypointense on T1 and hyperintense on T2-weighted images, reflecting their water content (fluid, oedema, etc.). Haemorrhage or infection causes different and heterogeneous signal intensities both on T1 and T2, depending on the age of the bleed or the protein concentration, making difficult the characterization of lesions.

**Figure 11:**
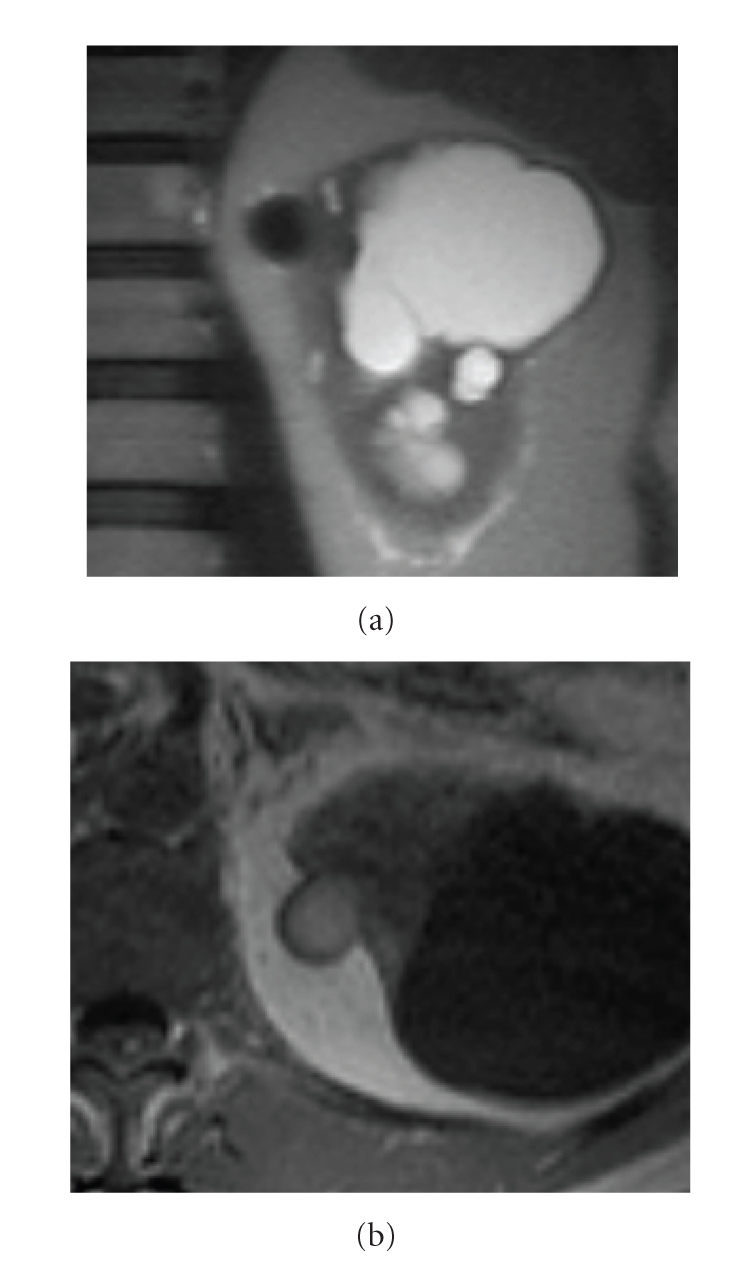
(a) Coronal haste T2 and (b) axial precontrast T1: small lesion on medial aspect of the upper pole of the left kidney shows a low signal intensity on T1 and hyperintensity on T1, revealing blood breakdown products consistent with haemorrhagic cyst.

In some cases, T1 hyperintensity and T2 hypointensity renal lesions are noticed (see Figure 11), and most of them are also hyperattenuating renal masses on unenhanced CT. These are either benign or malignant masses and include blood breakdown products or proteinaceous cysts, haematomas and vascular malformations, and oncocytomas or angiomyolipomas with minimal fat, but also some malignant lesions such as RCC and lymphomas. Shinmoto et al. [57] reported that papillary RCC is associated with T2-hypointense appearance as well as hemosiderin deposition, haemorrhage, and necrosis. When a solid hyperattenuated renal mass is seen on unenhanced CT, an MR must be performed to characterize the lesion looking for signal loss on T2-weighted images owing to blood products, iron deposits, hypercellularity, and proteinaceous content. MR is helpful to recognize lesions that are otherwise impossible to differentiate by CT alone, such as AML with minimal fat and clear cell renal cell carcinomas. However, differentiation between AML with minimal fat and papillary RCC is often not always possible by MR, and a percutaneous biopsy may be useful [42]. When a cystic mass is evaluated, MR may show additional thickness of the septa and wall on T2-weighted images than on CT [29].

#### 5.3.2. Presence of fatty tissue

**Figure 12:**
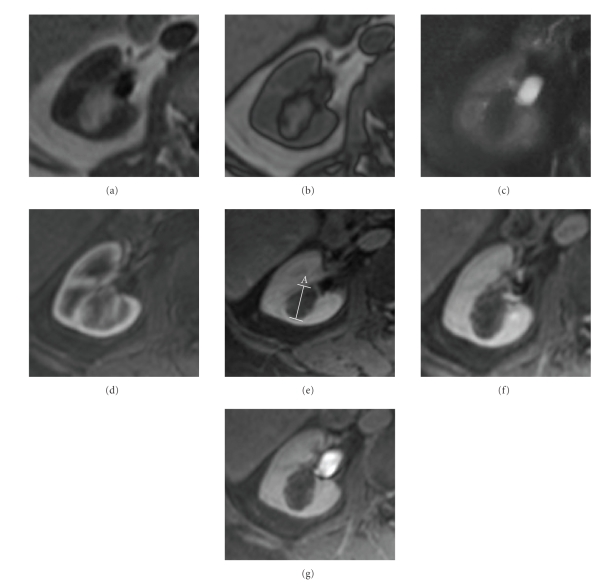
(a) Axial T1 in phase, (b) axial T1 out of phase, (c) axial haste T2 with fat saturation, and ((d–g) axial dynamic postcontrast T1 (d), arterial; (e), venous; (f), nephrographic, and (g), excretory phase): right yuxtahilar renal lesion seen as a T1 hyperintense mass that shows gross fat suppression on T2 revealing macroscopic fat and also probably small foci of peripheral fat as there is a small amount of signal loss on out of phase sequence. On dynamic postcontrast images the mass shows a transitional highly peripheral enhancement in the non-fat components with poor enhancement on the remaining postcontrast study. An angiomyolipoma was found at surgery.

AMLs are the only solid renal tumors that can be positively characterized using MR by demonstrating macroscopic fat in the lesion, and this fact is basically useful in patients with tuberous sclerosis, since they develop AML and an increasing risk of renal cell carcinomas [49] (see Figure 12).

Opposed phase images and spectral fat suppression are useful in differentiating fat from haemorrhage containing carcinomas causing high signal intensity on T1. Furthermore, in phase gradient echo images are often helpful because the fatty portions of AML will be hyperintense and renal clear cell carcinomas usually will not, although renal cell carcinomas incidentally may be hyperintense on T1; in these cases spectral fat suppression images should be used to prove the presence of macroscopic fat in the AML [49].

Some renal lesions may contain very small amount of fat, the so called “minimal fat AML,” with microscopic fat and without demonstrable macroscopic fat (angiomyomas), and it is not possible to differentiate from a renal neoplasm. Fat suppression techniques generally are not helpful for detecting fat in AML with minimal fat, because such masses contain little or no fat and often appear as isointense to the renal parenchyma. Chemical shift imaging may be used to determinate a small amount of fat within a mass, and can be used to differentiate AML with minimal fat from other renal neoplasms, with a high sensitivity and specificity [58].

However, a renal mass that is suppressed focally or diffusely on opposed-phase sequences and that does not exhibit fat suppression should arouse suspicion about the possible presence of clear renal cell carcinoma due to intracellular lipid [42]. Obviously, this differentiation cannot be made only with this unique technique and information of other sequences, such as signal intensity on T2 and dynamic gadolinium enhancement, and proper followup should therefore take place. Kim et al. [58] suggested followup with CT or MR imaging for two years after detection for such lesions.

The presence of fat in clear cell carcinoma has been used to differentiate subtypes of renal cell carcinoma because this fact does not occur in oncocytoma and transitional cell carcinoma [49], but if the tumor does not show signal loss, it can still be a clear cell carcinoma.

#### 5.3.3. Presence of pseudocapsule

This is a pathologic feature composed of fibrous tissue and compressed renal parenchyma, seen frequently in the early stages of a SRM. Although not specific (also seen in some oncocytomas), it has been related to renal cell carcinomas usually small and of low histologic grade, slow growing, and less likely to metastasize [15]. Their presence is an indicator of prognostic value [59]. This condition allows renal parenchyma-sparing surgery, especially if simple enucleation is considered in patients with multiple tumors, Von Hippel-Lindau disease or familial tendency for RCC.

**Figure 13:**
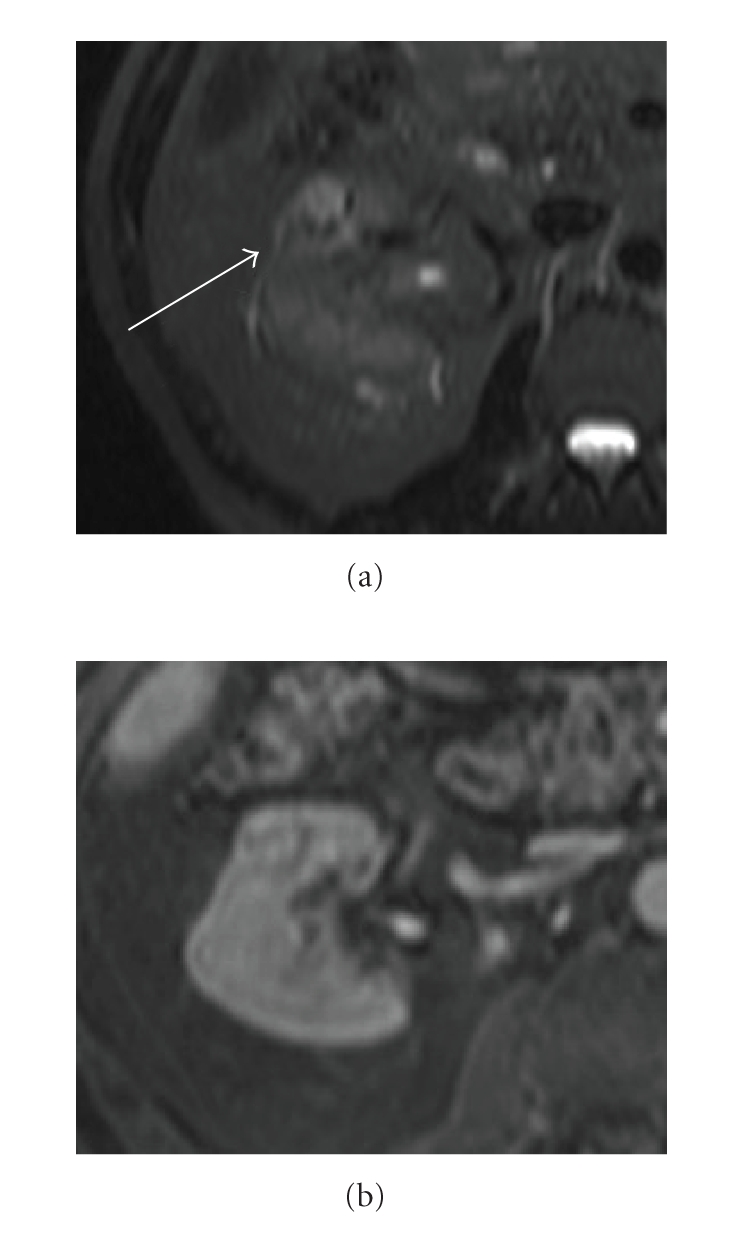
(a) Axial stir and (b) axial postcontrast T1. A pseudocapsule is seen as a hypointense rim surrounding the tumor on T2 and is better delineated than on postcontrast T1 owing to the late contrast enhancement of the pseudocapsule.

At MR, a pseudocapsule was seen as a hypointense thin rim surrounding the tumor on both T1- and T2-weighted images and is more difficult to detect in hypointense tumors [60, 61]. With postcontrast images, late enhancement of the pseudocapsule resulted in poor contrast relative to the surrounding tissue, lessening its own visualization in this sequence (see Figure 13). Some reports [61, 62] noticed that the presence of a pseudocapsule offers an additional value for local staging. On that series, T2-weighted imaging was the most sensitive technique for visualization of the pseudocapsule (sensitivity: 68%; specificity: 91%), and is corroborated by other authors [15, 63, 64]. For this reasons, MR shows a moderate to high sensitivity in depicting the pseudocapsule than CT [49]. In some large tumors, although tumor invasion was seen, a residual pseudocapsule was found in some areas.

#### 5.3.4. Involvement of perinephric fat

This is a key point in treatment planning in modifying the surgical approach from conservative to radical nephrectomy (Robson's stage I versus stage II). If partial nephrectomy is considered, it is essential to know preoperatively if the perinephric fat is invaded or not by the tumor. Although MRI appears slightly more sensitive than CT, it is not specific in distinguishing between these two stages.

The presence of an intact pseudocapsule is an indirect sign of lack of perinephric fat invasion [61]. The overall sensitivity of CT in detecting pseudocapsule is very low [62, 63, 65, 66], and MR had a pertinent accuracy for evaluating possible involvement of perinephric fat using the aspect of the pseudocapsule as an additional feature [61].

Perirenal fat invasion diagnosis was made when there was loss of capsular integrity indicated by interruption of the low signal intensity line around the kidney on T1- and T2-weighted images and thick (>0.5 mm) perinephric stranding. Thin perinephric stranding and collateral vessel formation alone were not considered features of perinephric fat invasion. This causes under- and overstaging on CT and MR and lacks good specificity [67]. It also may be caused by edema, vascular engorgement, and/or inflammation [65]. The only highly specific finding was the presence of an enhancing nodule or soft-tissue mass in the perinephric space but this sign had only 46% sensitivity [38].

#### 5.3.5. Enhancement

Different patterns of enhancement have been observed, predominantly peripheral, heterogeneous, and homogeneous. The dynamic evaluation appears to be useful in the detection and characterization of simple renal cysts and solid neoplasms [68].

**Figure 14:**
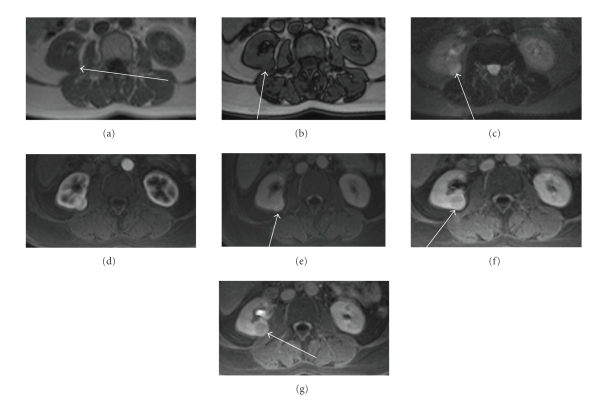
(a) Axial T1 in phase, (b) axial T1 out of phase, (c) axial stir, and (d–g) axial dynamic postcontrast T1 ((d) arterial; (e) venous; (f) nephrographic, and (g) excretory phase). A nonfatty mass hyperintense on T2 and highly vascular on corticomedullary and nephrographic phase with late mild washout as is shown on this clear renal cell carcinoma.

The presence or absence of enhancement within a renal mass is the most important factor in its proper characterization. When a predominant part of a renal mass enhances, the mass is considered solid and likely neoplastic. Otherwise, it is important to be aware of the possibility of pseudoenhancement and to know when to suspect it. All solid lesions demonstrated gadolinium enhancement, not only RCC and invasive transitional cell carcinomas but also oncocytomas and AML (see Figures 12 and 14). Although enhancement is sufficient for predicting malignancy, nonenhancement is not sufficient to exclude malignancy, and again the integration of T2 appearance is useful in improving the differentiation between benign from malignant renal lesions [41].

**Figure 15:**
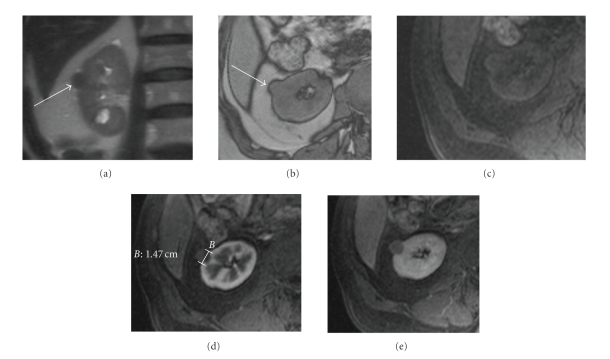
(a) Cor haste T2, (b) out of phase axial T1, (c) axial precontrast T1 with fat suppression, (d) early postcontrast axial T1 with fat saturation, and (e) axial postcontrast T1 with fat suppression. Small renal lesion on the lateral aspect of the upper pole of the right kidney shows a solid mass hypointense on T2 that does not present a loss of signal out of phase sequence and only a small enhancement on postcontrast sequences, consistent with a papillary renal carcinoma.

Hypervascular RCC can be easily differentiated on dynamic contrast-enhanced MR. Hypovascular RCC, AML, and complicated cysts enhanced significantly less than cortical and medullary tissue did (see Figure 15). Furthermore, papillary RCC is typically hypovascular and shows mild contrast enhancement, whereas AML with minimal fat is generally hypervascular and shows marked enhancement, but occasionally the degree of enhancement varies, making this differentiation difficult [42]. Also hypovascular RCC from the first minute after gadolinium injection showed significantly greater enhancement than complicated cyst [69].

Thus, areas with haemorrhage or infection products do not enhance but their signal intensity remains higher than that of simple cyst, making quantitative ROI measurements in these lesions essential [70] for correct characterization. This can be made by quantitative and qualitative methods. Ho et al. [71] concluded that above 15% was the optimal percentage of enhancement threshold for distinguishing cysts from malignancies. Although usually quantitative and qualitative methods are sensitive in the detection of enhancement, in hyperintense lesions on unenhanced T1, qualitative assessment based on image subtraction should be performed to avoid false negative quantitative results [54].

**Figure 16:**
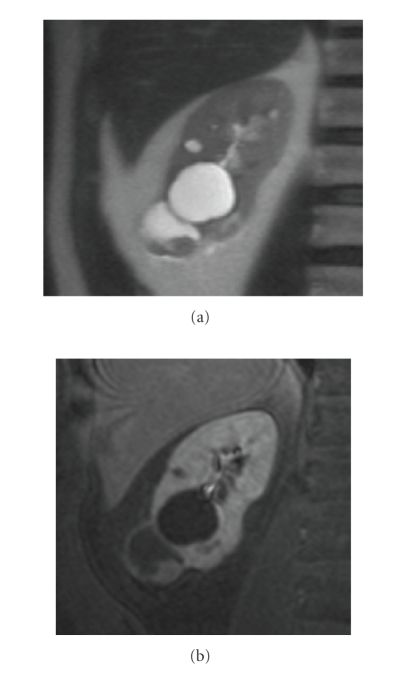
(a) Coronal haste T2 and (b) late postcontrast coronal T1 with fat saturation. Cystic lesion with an enhancing nodule on the caudal aspect of the kidney. Renal cell carcinoma was found at surgery.

When a cystic mass is evaluated, MR imaging may demonstrate definitive enhancement that shows only equivocal enhancement on CT. In a cystic lesion with only a small solid component, subtraction images may again be used to better assess the presence of enhancement [49]. Furthermore, even if detecting a calcification is a limitation on MR imaging, it is however an advantage to determine whether enhancement is present in a heavily calcified cyst on CT given that it could be better appreciated [45]. The combination of mural irregularity and intense mural enhancement is a strong predictor of malignancy in renal cystic lesions (see Figure 16). However, the appearance of benign and malignant lesions may overlap [72, 73].

#### 5.3.6. Other

Invasion of Gerota's fascia was diagnosed when continuity of the low signal intensity line around the perinephric fat on T1-weighted images was disrupted by tumor. Imaging of the ipsilateral adrenal gland and venous spread of tumors are out of the scope of this chapter, thus the clinical setting of small renal tumors is not found incidentally, as it is the case in the low probability of nodal metastases in this stage of disease.

On the other hand, advanced imaging techniques led to improve the global accuracy for MDCT to adequately stage these clinical aspects [38]. Hricak et al. [60] reported accuracy rates for detecting adjacent organ invasion with MRI, although, in their series, overstaging was caused by the presence of abnormal signal, indistinct interface, and absence of a free fat plane between the tumor and the adjacent organ.

### 5.4. Interpretation of images and surgical criteria

When characterizing a renal mass, the major question to be answeredis whether the mass represents a surgical or nonsurgical lesion or, in some cases, if followup studies are necessary.

Magnetic resonance imaging allows an accurate differentiation between solid and cystic masses, as a first approximation, but angiomyolipomas are the only solid renal tumors that can be positively characterized using MR. Nevertheless, Prasad et al. [74] reported that small renal medullary tumors may be differentiated from the more common renal adenocarcinomas by their central location and certain demographic characteristics. Furthermore, some authors recently [75] analyzed the correlation between MR image features and histopathological findings, giving value to subvoxel fat on chemical shift imaging as a good correlation to clear cell type with a high specificity. These authors reported that small size, peripheral location, low intratumoral signal intensity on T2, and low level enhancement were also associated with low-grade papillary carcinomas.

Detection of macroscopic *fat* is the key for diagnosing AML in the proper clinical setting, and it is decisive because AML does not need to be surgically removed. The diagnosis is made by demonstrating fat within a solid renal mass. One pitfall of fat containing renal masses is the presence of a renal cell carcinoma involving the perinephritic fat. This differentiation is easily made if there is some calcification on CT, as do some RCC [29]. However, calcium is not always present in renal cell carcinoma [76].

The observation of a *pseudocapsule* surrounding a renal cell carcinoma is a sign of lack of perinephric fat invasion, and therefore it is more likely to predict that the tumor can be removed by nephron sparing, so partial nephrectomy or simple enucleation may be indicated when a pseudocapsule is detected [49, 62].

The presence of *haemorrhagic* products may obscure enhancement on dynamic postcontrast T1 images, and it may also contribute to heterogeneity on T2 images. Thus, in the setting of a T2 heterogeneous nonenhancing mass, careful followup after an antibiotic trial may be a prudent recommendation to avoid nephrectomy of renal abscess and to avoid misdiagnosis of a hemorrhagic renal cell carcinoma [41]. Furthermore, a common occurrence of haemorrhage is described in patients with renal cancer and/or in patients with renal insufficiency, and caution should be exercised when evaluating haemorrhagic cystic lesions in these patients [77].

The most importantcriterion used in differentiating surgical fromnonsurgical renalmasses is the determination of *enhancement* [29]. Despite that, the lack of enhancement of a renal lesion, particularly if small (<1 cm), is not considered a sufficient criterion for excluding malignancy, as the haemorrhagic lesions occur, and in this setting, T2-weighted images must be considered. Moreover, it is important to combine the degree of enhancement with the morphologic features of the lesion, such as homogeneity, wall thickening, and presence of calcifications.

When a *cystic* mass is evaluated, a surgical cyst can be suspected only if enhancement is present. Calcification in a cystic renal mass is not as important in diagnosis as the presence of associated enhancing soft-tissue elements [78]. MR imaging may depict additional septa, thickening of the wall and/or septa, or enhancement, which may lead to an upgraded Bosniak cyst classification and can affect case management [31, 49].

### 5.5. Limitations

As with all imaging techniques, it is extremely difficult with MR to determine whether malignant tissue extends to adjacent normal tissue when strictly regular margins are found because microscopic local invasion could have occurred [61, 64]. Staging errors were made because of limitations of the imaging technique: inability to detect microscopic invasion of the perinephric fat, difficulty in differentiating inflammatory changes from tumor infiltration, and insensitivity in differentiating small collateral blood vessels from tumor extension in the lymphatics [67].

There are also limitations to the detection of a pseudocapsule by MR, mainly with hypointense tumors because its detection may be less accurate on T2-weighted owing to the lack of delineation of the surrounding rim, as is the case of some papillary tumors. With SRM, partial volume averaging may obscure its visualization, thus evaluation in three planes, and coronal, sagittal, or oblique views are required to avoid this phenomenon on the upper and lower part of the tumor [61]. Furthermore, given that the pseudocapsule was also found in oncocytomas, it is not useful for differentiating RCC from this benign solid tumor, and it cannot be used to predict the nature of the lesion. But it can offer an additional value to the performance of preoperative MR to stage renal tumors, aiding to make decisions about the most appropriate surgical technique to employ.

Concerning nodal staging, MR has the same limitations as CT according to the nodal size over 1 cm in short-axis diameter, being this an additional indication for the future use of new iron oxide-based contrast agents on MR to improve specificity and accuracy in nodal staging [49, 79].

## Figures and Tables

**Table 1:** Staging system for renal carcinoma and CT criteria.

Tumour position	Robson	TNM	CT findings
*Confined within *	**I**		Soft-tissue mass enhances
*renal capsule*			less than normal renal
* *Small (<7 cm)		**T1**	parenchyma; central
* *Large (≥7 cm)		**T2**	necrosis in large RCC.

			Perinephric stranding;
*Spread to perinephric fat*	**II**	**T3a**	Perinephric collateral vessels;
			Soft-tissue mass in perinephric space

*Venous thrombus*	**III A**		
* *Renal vein only		**T3b**	Low-attenuation filling defect vein;
* *IVC infradiaphragmatic		**T3c**	Direct continuity of thrombus with primary mass;
* *IVC supradiaphragmatic		**T4b **	Enhanced thrombus

*Regional lymph *	**III B**	**N1-N3**	Lymph nodes 1 cm in diameter or larger
*node metastases*			

*Direct invasion of adjacent organs*	**IV A **	**T4a**	Obliteration of normal soft-tissues planes between tumor and adjacent organs

*Distant metastases *		**M1**	Metastases enhance with IV contrast material;
			Hepatic metastases best in arterial phase

IV: intravenous, IVC: inferior vena cava.
